# Severe asthma as the initial clinical manifestation of IgG4-related disease: a retrospective clinical study

**DOI:** 10.1186/s12890-022-01937-9

**Published:** 2022-04-12

**Authors:** Xiangning Liu, Chi Shao, Chen Yu, Hui Huang, Ruili Pan, Kai Xu, Xin Zhang, Zuojun Xu

**Affiliations:** 1 Department of Internal Medicine, Peking Union Medical College Hospital, Chinese Academy of Medical Sciences and Peking Union Medical College, No.1 Shuaifuyuan Street, Dongcheng District, Beijing, 100730 China; 2 Department of Pulmonary and Critical Care Medicine, Peking Union Medical College Hospital, Chinese Academy of Medical Sciences and Peking Union Medical College, No.1 Shuaifuyuan Street, Dongcheng District, Beijing, 100730 China; 3 Radiological Department, Peking Union Medical College Hospital, Chinese Academy of Medical Sciences and Peking Union Medical College, No.1 Shuaifuyuan Street, Dongcheng District, Beijing, 100730 China; 4 Medical Records Department, Peking Union Medical College Hospital, Chinese Academy of Medical Sciences and Peking Union Medical College, No.1 Shuaifuyuan Street, Dongcheng District, Beijing, 100730 China

**Keywords:** Severe asthma, Immunoglobulin G4-related disease, Respiratory involvement, Eosinophilia

## Abstract

**Background:**

Respiratory involvement is common in immunoglobulin G4-related disease (IgG4-RD). However, severe asthma as the initial clinical manifestation of IgG4-RD is rare and might be neglected by respiratory clinicians. We aimed to explore the clinical characteristics and prognoses of patients with immunoglobulin G4-related disease (IgG4-RD) manifesting as severe asthma.

**Methods:**

A retrospective analysis of the clinical characteristics and prognoses of patients with severe asthma who were eventually diagnosed with IgG4-RD was performed in the Peking Union Medical College Hospital from 2013 to 2019.

**Results:**

Twelve patients (5males, 7 females) were included. The mean age at enrollment and age of asthma onset were 59.4 ± 10.1 and 53.8 ± 10.4 years, respectively. The mean duration of asthma symptoms was 5.7 ± 2.0 years. In all patients, the proportion (25.1 ± 10.3%) and count (2.0 ± 1.1) × 10^9^/L of eosinophils in peripheral blood increased. Additionally, all patients exhibited elevated total immunoglobulin E [IgE, (1279.3 ± 1257.9) KU/L] and IgG4 (9155.8 ± 9247.6) mg/dL. Bronchial wall thickening (n = 11) and mediastinal/hilar lymphadenopathy (n = 11) were major chest CT manifestations. All were pathologically diagnosed through surgical biopsy; submandibular gland (n = 8), supraclavicular lymph node (n = 2), stomach (n = 1), rashes (n = 1), lacrimal gland (n = 1) and thoracoscopic lung (n = 1) biopsies were performed. Asthma was well controlled by oral glucocorticoids (GCs), but some patients relapsed during tapering (n = 11). The refractory condition was controlled after increasing the dosage of GCs and add-on immunosuppressants.

**Conclusions:**

For patients with middle age-onset severe asthma with elevated eosinophils, total IgE and IgG4 levels and available salivary gland ultrasound imaging, ruling out IgG4-RD is recommended. GCs used in combination with immunosuppressants is recommended to prevent relapse.

## Background

Severe asthma greatly affects the quality of life and survival of these patients [1]. Accordingly, it is important to identify the underlying disease and inappropriate treatments to improve the prognosis of patients with severe asthma [1, 2]. Eosinophilic granulomatosis with polyangiitis (EGPA) and allergic bronchopulmonary aspergillosis (ABPA) are major differential diagnoses of adult severe asthma patients with elevated eosinophils [1]. However, asthma-like manifestations are not rare in immunoglobulin G4-related disease (IgG4-RD), and approximately 30% of patients with IgG4-RD will show elevated peripheral eosinophils and serum IgE levels [3].

IgG4-RD is a rare systemic autoimmune disease that has been gradually recognized and reported since 2003. Common clinical manifestations include autoimmune pancreatitis, Mikulicz disease, retroperitoneal fibrosis [3]. Nevertheless, IgG4-RD tends to be ignored by physicians when asthma is the clinical manifestation at onset. In this study, a retrospective analysis of the clinical-radiological-pathological characteristics of 12 patients with IgG4-RD whose initial manifestation was severe asthma was carried out to draw attention and to improve our understanding of this disease.

## Materials and methods

### Study design

Patients who met the following criteria were included: (1) adult (age ≥ 18 years old); (2) pathologically diagnosed with IgG4-RD from January 2013 to December 2019 at the clinics of the Department of Pulmonary and Critical Care Medicine at Peking Union Medical College Hospital (PUMCH); (3) severe asthma was the initial diagnosis or main complaint; and (4) regularly followed up at our clinics and with complete follow-up data.

### Definitions of severe asthma and IgG4-RD

According to the 2014 International ERS/ATS guidelines [1], severe asthma is defined as asthma requiring treatment with medications of steps 4–5 by the Global Initiative for Asthma (GINA)’s recommendation for the previous year, i.e., high-dose inhaled corticosteroids and a long- acting β2-agonist (LABA), or leukotriene modifier/theophylline, or continuous or near continuous systemic glucocorticoids (GCs) for more than half of the previous year to avoid an “uncontrolled” status or to avoid remaining “uncontrolled” despite these medications. Patients with controlled asthma who were suffered from more than one exacerbation after tapering of these high doses of inhaled corticosteroids or systemic GCs were also diagnosed with severe asthma.

The diagnosis of IgG4-RD was confirmed according to relevant diagnostic criteria in 2011 [4, 5]: a. at least two pairs of classic organs with symmetrical swelling for more than 3 months; b. elevated serum IgG4 (> 1350 mg/L); and c. histopathological features including lymphocyte and IgG4^+^ plasma cell infiltration (IgG4^+^ plasma cells/IgG^+^ plasma cells > 50%, and > 10 IgG4^+^ plasma cells/high power field) with typical tissue fibrosis or sclerosis. Diagnosis was confirmed by meeting all of the above criteria and ruling out malignancies, other systematic connective tissue diseases, and other diseases that mimic IgG4-RD. In our study, the “classic organs” included lacrimal, parotid, submandibular, sublingual glands, and some minor salivary glands. The swelling of the classic organs was identified by color Doppler ultrasonography. “Sclerosis” was identified via tissue biopsy by pathologists. The detailed pathological features of typical tissue fibrosis or sclerosis were in accordance with the “explanatory notes” of comprehensive diagnostic criteria for IgG4-RD [5].

### Data collection

Data including demographics, serum biomarkers, chest CT imaging and histopathologic findings were extracted from electronic medical records. The blood for the all subsequent laboratory analyses was drawn at the time when the patient was diagnosed with severe asthma and they were suspected to have secondary asthma. It had been at least 8 weeks since the most recent systemic GC administration. This study was approved by the institutional review board of PUMCH (No. JS-2517). Informed consent was signed by the patients or their authorized family members.

### Statistical analysis

Continuous values are expressed as the mean ± standard deviation (SD) or range, and categorical data are reported as the number of patients (percentage). SPSS (v. 21.0, SPSS Inc., Chicago, IL, USA) was used for data analysis.

## Results

### Clinical characteristics of enrolled patients

Twelve of 14 patients were enrolled in our study; two patients were excluded because they were lost to follow-up after diagnosis, or no chest CT imaging was available. The general information about the 12 enrolled patients is summarized in Table 1. Five of the 12 included patients were male. The mean age was 59.4 ± 10.1 (range 41–75) years, the mean age at asthma onset was 53.8 ± 10.4 (range 38–70) years, and the mean duration of severe asthma was 5.7 ± 2.0 (range 3–10) years before a final diagnosis of IgG4-RD was confirmed.

**Table 1:** General clinical characteristics for all enrolled cases

Case	Gender	Age (y)	Onset age (y)	Eos%/n^*^ (%/10^9^/L)	Eos(n)^#^ (10^9^/L)	T-IgE^*^ (KU/L)	T-IgE^#^ (KU/L)	IgG (g/l)	IgG4^*^ (mg/dl)	IgG4^#^ (mg/dl)	Biopsic tissues	Treatment	Outcomes
1	F	57	48	18.8/1.09	0.11	1378	257	23.2	17,100	5430	Submandibular gland	GCs + TII	Refractory
2	F	56	52	40.5/3.86	0.11	2053	1311	11.52	4860	2160	Stomach + supraclavicular LN	GCs + CTX	Refractory
3	F	74	69	41.5/4.23	0.4	1626	115	12.67	1560	670	Skin + supraclavicular LN	GCs + CTX	Refractory
4	M	41	38	20.4/1.29	0.11	381	12	32.72	4020	4020	Submandibular gland	GCs + TII	Refractory
5	F	60	53	35.4/2.78	0.2	448	48	15.4	1570	780	Submandibular gland	GCs + TII	Refractory
6	F	51	46	15.6/1.45	0.3	326	186	11.5	7110	2110	Lacrimal gland	GCs + CTX	Refractory
7	M	67	63	17.1/1.53	0.3	4681	345	37.43	4890	2650	Submandibular gland	GCs + CTX	Refractory
8	M	53	43	34.3/2.1	0	2485	266	35.3	26,900	1970	Submandibular gland	GCs + CTX	Refractory
9	M	72	67	9.9/0.8	0.2	460	153	12.3	3590	2370	Submandibular gland	GCs	Cured
10	F	56	51	27.9/2.19	0.1	284	57	22.11	7490	615	Submandibular gland	GCs + CTX	Refractory
11	F	51	45	25.3/1.75	0.2	239	92	14	2080	243	Submandibular gland	GCs + CTX	Refractory
12	M	75	70	14.2/0.98	0.3	991	354	28.9	28,700	6400	Lung	GCs + CTX	Refractory

*F* female, *M* male, *Eos* eosinophils, *Ig* immunoglobulin, *GC* glucocorticoids, *LN* lymph node, *TII* *Tripterygium wilfordii*, *CTX* cyclophosphamide

*Before systemic glucocorticoids administration, ^#^three months after the systemic glucocorticoids administration

All 12 of the patients had cough and recurrent episodes of wheezing; 9 had clear sputum, and 3 had recurrent low fever, but there was no hemoptysis. There were 10 cases of recurrent allergic rhinitis, 9 cases of salivary gland ultrasound abnormalities (including swelling and abnormal echo manifestations), 3 cases of lacrimal gland swelling, 3 cases of dry mouth, and 2 cases of rash.

### Serum biologic markers

Regarding the complete blood count (CBC) analysis, one patient had an elevated white blood cell count (10.2 × 10^9^/L) and four patients had a decreased lymphocyte proportion (< 20%). All patients had elevated proportions (25.1 ± 10.3%, range 9.9–41.5%) and counts (2.0 ± 1.1 × 10^9^/L, range 0.8–4.23 × 10^9^/L) of eosinophils in their peripheral blood. The platelet count decreased in one patient (41 × 10^9^/L). All patients had normal hemoglobin levels.

Regarding total IgE, IgG, and IgG subtypes, all patients had elevated serum total IgE (1279.3 ± 1257.9 KU/L, range 239–4681 KU/L) and IgG4 (9155.8 ± 9247.6) mg/dl (range 1570–28,700 mg/dl). The mean serum IgG was 21.4 ± 9.5 g/L, and 6 patients had hyperglobulinemia (> 17 g/L).

Miscellaneous findings were as follows. All 12 patients were negative for antineutrophil cytoplasmic antibodies, *Aspergillus fumigatus*-specific antigen (m3) and a variety of other *Aspergillus*-specific antigen complexes (m2x). Seven patients had elevated C-reactive protein levels. Only four patients underwent serum complement analysis, with one having decreased levels. For all patients, pulmonary function tests showed obstructive ventilatory dysfunction, with confirmed reversible airflow limitation. The mean fraction of NO in exhaled air (*F*_eNO_) was [(61.1 ± 24.8) ppb, range (30–101) ppb], a significant elevation.

### Chest CT imaging and pathologic features

**Fig. 1:**
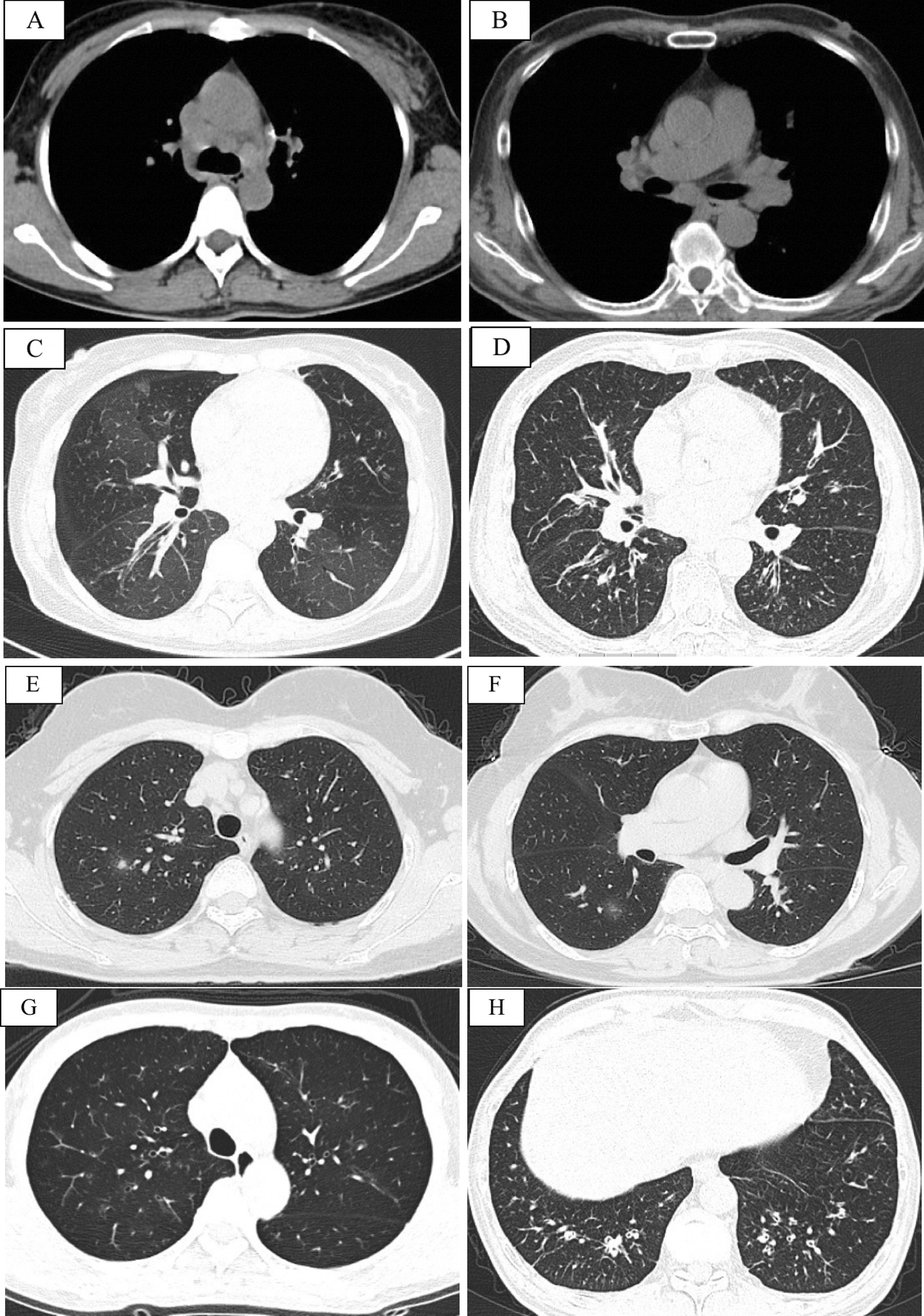
**A**–**H** Chest CT features for the enrolled cases before administration with systemic glucocorticoids: mediastinal lymphadenopathy (**A** and **B**), mosaic attenuation sign (**C**), diffuse bronchial wall thickening (**D**), scattered solid or ground-glass nodule (**E** and **F**, respectively), diffuse peribronchiolar ground-glass opacities (**G**), and multiple bronchiectasis (**H**)

All patients underwent chest high-resolution CT (HRCT). Based on the mediastinal window, 11 patients had mediastinal/hilar LN enlargement (shorter diameter > 1 cm), although no pleural or pericardial effusion was detected. Based on the lung window, 11 patients had multiple bronchial wall thickenings in different lung areas, 6 had scattered ground-glass opacities, and 2 had solid lung nodules. (Fig. 1A–H).

All patients underwent surgical biopsy, including submandibular gland (n = 8), supraclavicular LN (n = 2), stomach (n = 1), rashes (n = 1), lacrimal gland (n = 1) and thoracoscopic lung (n = 1) biopsies. Two patients had biopsies performed on two sites, one patient for stomach and supraclavicular LN and the other patient for skin and supraclavicular LN. All pathological findings were in accordance with the microscopic manifestations of IgG4-RD [4, 5].

### Treatments and outcomes

All patients received systemic glucocorticoids (GCs) after the diagnosis of IgG4-RD: the initial dose was equivalent to 0.8–1.2 mg/kg/day prednisone and was reduced after 2–3 weeks. Eleven patients experienced different extents of relapse after GCs were reduced to prednisone at 10–20 mg per day. Intensive treatments were administered after relapse, including increasing doses of oral GCs and/or combining GCs with immunosuppressive agents (8 patients received cyclophosphamide per os and 3 patients received *Tripterygium wilfordii*), were given after relapse. Remission was achieved after intensive treatments in all patients. Furthermore, all patients received inhaled GCs and long-acting bronchodilators when oral prednisone was less than 15 mg per day.

In all patients, the proportion of eosinophils in the peripheral blood returned to normal after symptoms were controlled with systemic GCs. Although their symptoms disappeared with treatment, serum levels of IgG4 and total IgE returned to normal ranges in only 4 and 3 patients, respectively.

## Discussion

With the extensive administration of inhaled GCs and bronchodilators and the implementation of patient education, the number of adult patients with severe asthma has decreased significantly. However, the condition is still an important factor leading to poor outcomes and even death, attracting much attention [1, 2, 6]. The 2014 ERS/ATS guidelines on severe asthma indicate that severe asthma with elevated peripheral blood eosinophils should be differentiated from hypereosinophilic syndrome, allergic pneumonia, EGPA or ABPA [1]. Our study shows that IgG4-RD can also manifest as severe asthma with elevated peripheral eosinophils. Such patients may have significantly elevated levels of IgG4 and total IgE, salivary gland involvement, mediastinal/hilar lymphatic enlargement and bronchial wall thickening on chest CT imaging. Although IgG4-RD can be well controlled by systemic GCs, it easily relapses when GCs are tapered. IgG4-RD should also be listed as hypereosinophilic severe asthma, and biopsy of the involved organ(s) is vital for the final diagnosis.

With the growing knowledge of IgG4-RD [5–8], respiratory system involvement was found to be common, occurring in approximately half of IgG4-RD patients from 2001 to 2019 [7, 9–12]. IgG4-RD can involve various thoracic tissues and organs, such as the airway, pleura, lung, and intrathoracic LNs. As a result, clinical phenotypes differ among patients [7, 10, 12–22]. Indeed, since Ito et al. [23] reported that IgG4-RD can manifest as asthma, there have been other reports on asthma and IgG4-RD [13, 16, 24–26].

IgG4-RD is more common in males (M:F = 1.6:1) and in middle-aged or elderly patients [3], whereas refractory asthma may have a younger age of onset. According to the study by Bulow et al. [2], the mean age of disease onset is approximately 25 years, and the majority of patients with severe asthma are female (59%). However, in our cohort, the average age of disease onset was 53.8 ± 10.4 years, and there were more female patients (58.3%). Clinically, rather than solely considering severe asthma, it is necessary to be aware of the possibility of IgG4-RD and other systemic diseases manifesting as severe asthma in middle-aged and elderly people.

The head and neck, especially the eyes and salivary glands, are common sites of involvement in IgG4-RD (80%) [3]. In our study, 8 patients were diagnosed based on submandibular gland biopsies. Mikulicz’s disease, namely, IgG4-related lacrimal gland inflammation and sialadenitis, is a special phenotype of IgG4-RD [3, 27]; it mainly manifests as symmetrical lacrimal or salivary gland enlargement, and biopsies show typical pathological findings of IgG4-RD. Matsui et al. reported that 28% of patients with Mikulicz’s disease develop asthma, and according to chest CT, mediastinal/hilar lymphadenopathy (52%) is the most common radiological manifestation. Patients with lung involvement of Mikulicz disease have higher levels of serum total protein, IgG, IgG4, and IgE than patients with only lacrimal or salivary involvement [13]. Of note, Baqir et al. [16] paid special attention to the relationship between periorbital lesions and asthma in patients with IgG4-RD: 16 of 31 patients had asthma (52%), and their median age of asthma onset was 56 years old; patients with asthma had higher levels of serum IgG4; and the most common chest CT abnormalities were mediastinal/hilar LN enlargement and bronchial vascular bundle thickening. Furthermore, Ito et al. [23] reported that patients with IgG4-related autoimmune pancreatitis manifesting as asthma also had high levels of serum IgG, IgG4, and IgE. In our study, all patients had elevated proportions and counts of eosinophils, elevated IgG4 and elevated total IgE. However, only 50% of the patients had hyperglobulinemia, and many of them had elevated IgG1 and IgG2. Bronchial wall thickening occurred in 91.7% of the patients in our study, and it is considered to be associated with refractory severe asthma. Nevertheless, our study also found that as many as 91.7% of patients exhibited mediastinal/hilar lymphadenopathy, which should arouse the attention of respiratory clinicans who normally work with severe asthma patients. These manifestations may be caused by the different clinical phenotypes between our patients and other reported IgG4-RD cases. However, sarcoidosis, mycobacterial infection, and other kind of malignancy were the common causes of mediastinal/hilar LN enlargement and dyspnea. Endobronchial ultrasound-guided transbronchial needle aspiration (EBUS-TBNA) is an important examination for the differentiation. Overall, hypergammaglobulinemia, salivary gland or periorbital involvement, and mediastinal/hilar lymphadenopathy on chest CT might be the risk factors for an asthma-like phenotype in IgG4-RD.

For the vast majority of patients with IgG4-RD, symptoms were well controlled by systemic GCs. However, patients with multiple organ/system involvement and elevated levels of IgE, IgG4, and eosinophils were prone to experience relapse during GC tapering [27]. Maintenance therapy with GCs combined with immunosuppressive or biological agents, e.g., rituximab, is generally required [16, 28, 29]. The recurrence rate in our study was as high as 91.7%, which may be related to factors such as multisystem involvement and high IgE, IgG4, and eosinophil levels at diagnosis. Our study also found that regardless of symptom improvement, disease remission, and normal eosinophil levels with systemic GCs, serum total IgE and IgG4 levels remained elevated in most patients. Because of the small number of patients in our study, it remains unclear whether the abnormality of these biomarkers was related to disease relapse after GC reduction.

This study has several limitations. First, as the selected patients all had refractory asthma, elevated eosinophils, and pathologically confirmed IgG4-RD, selection bias existed. Second, as IgG4-RD is a rare disease, the sample size was small, even though refractory asthma occurs in 3–10% of patients with asthma [6]. Nonetheless, approximately 40% of patients with IgG4-related autoimmune pancreatitis experience allergies, but fewer than 30% of patients present with asthma [16]. Third, we did not routinely screen the diagnosis of IgG4-RD for all severe asthma patients. Pulmonary function tests and/or asthma-associated symptoms are not usually consulted for IgG4-RD patients. It is expected that further studies will screen severe or relapsing asthma patients for IgG4-RD, and perform pulmonary function tests for Mikulicz’s disease or for IgG4-RD patients who were complained with cough or dyspnea etc.

## Conclusions

In conclusion, for middle-aged patients with severe asthma, elevated eosinophils and total IgE levels, it is recommended that serum IgG4, salivary gland ultrasonography and biopsy be performed to diagnose IgG4-RD. Once the diagnosis is confirmed, GCs combined with immunosuppressants are recommended.

## Data Availability

All data generated or analyzed during this study are included in this published article. Besides, any additional data/files may be obtained from the corresponding author on reasonable request.

## Abbreviations

IgG4-RD: Immunoglobulin G4-related disease
EGPA: Eosinophilic granulomatosis with polyangiitis
ABPA: Allergic bronchopulmonary aspergillosis
PUMCH: PEKING Union Medical College Hospital
GINA: Global Initiative for Asthma
GCs: Glucocorticoids
CBC: Complete blood count
m3: *Aspergillus fumigatus*-Specific antigen
m2x: *Aspergillus*-Specific antigen complexes
*F*_eNO_: NO in exhaled air
HRCT: High-resolution CT
LN: Lymph node

## References

[1] Chung KF, Wenzel SE, Brozek JL, Bush A, Castro M, Sterk PJ (2014). International ERS/ATS guidelines on definition, evaluation and treatment of severe asthma. Eur Respir J.

[2] von Bulow A, Backer V, Bodtger U, Søes-Petersen NU, Vest S, Steffensen I (2018). Differentiation of adult severe asthma from difficult-to-treat asthma—outcomes of a systematic assessment protocol. Respir Med.

[3] Lanzillotta M, Mancuso G, Della-Torre E (2020). Advances in the diagnosis and management of IgG4 related disease. BMJ.

[4] Zhang PP, Zhao JZ, Wang M, Feng RE, Liu XW, Lai XM (2017). The clinical characteristics of 346 patients with IgG4-related disease. Zhonghua Nei Ke Za Zhi.

[5] Umehara H, Okazaki K, Masaki Y, Kawano M, Yamamoto M, Saeki T (2012). Comprehensive diagnostic criteria for IgG4-related disease (IgG4-RD), 2011. Mod Rheumatol.

[6] Israel E, Reddel HK (2017). Severe and difficult-to-treat asthma in adults. N Engl J Med.

[7] Campbell SN, Rubio E, Loschner AL (2014). Clinical review of pulmonary manifestations of IgG4-related disease. Ann Am Thorac Soc.

[8] Wallace ZS, Naden RP, Chari S, Choi HK, Della-Torre E, Dicaire JF (2020). The 2019 American College of Rheumatology/European League Against Rheumatism classification criteria for IgG4-related disease. Ann Rheum Dis.

[9] Fei YY, Shi JH, Lin W, Chen Y, Feng RE, Wu QJ (2015). Intrathoracic involvements of immunoglobulin G4-related sclerosing disease. Medicine (Baltimore).

[10] Ryu JH, Sekiguchi H, Yi ES (2012). Pulmonary manifestations of immunoglobulin G4-related sclerosing disease. Eur Respir J.

[11] Corcoran JP, Culver EL, Anstey RM, Talwar A, Manganis CD, Cargill TM (2017). Thoracic involvement in IgG4-related disease in a UK-based patient cohort. Respir Med.

[12] Zen Y, Inoue D, Kitao A, Onodera M, Abo H, Miyayama S (2009). IgG4-related lung and pleural disease: a clinicopathologic study of 21 cases. Am J Surg Pathol.

[13] Matsui S, Taki H, Shinoda K, Suzuki K, Hayashi R, Tobe K (2012). Respiratory involvement in IgG4-related Mikulicz's disease. Mod Rheumatol.

[14] Srivali N, Ratanapo S, Ungprasert P, Cheungpasitporn W (2013). Significance of lymphadenopathy in IgG4-related sclerosing disease and sarcoidosis. Chest.

[15] Ogoshi T, Kido T, Yatera K, Oda K, Nishida C, Yamasaki K (2015). Incidence and outcome of lung involvement in IgG4-related autoimmune pancreatitis. Respirology.

[16] Baqir M, Garrity JA, Vassallo R, Witzig TE, Ryu JH (2016). Asthma and orbital immunoglobulin G4-related disease. Ann Allergy Asthma Immunol.

[17] Han GJ, Hu H, Mao D, Bai X, She DY, Zhao SF (2017). IgG4-related lung disease: analysis of 8 cases and literature review. Zhonghua Jie He He Hu Xi Za Zhi.

[18] Wu M, Wang L, Shang GG, Hong QY (2018). Clinical analysis of IgG4-related lung disease. Zhonghua Yi Xue Za Zhi.

[19] Morales AT, Cignarella AG, Jabeen IS, Barkin JS, Mirsaeidi M (2019). An update on IgG4-related lung disease. Eur J Intern Med.

[20] Matsui S (2019). IgG4-related respiratory disease. Mod Rheumatol.

[21] Moura MC, Gripaldo R, Baqir M, Ryu JH (2020). Thoracic involvement in IgG4-related disease. Semin Respir Crit Care Med.

[22] Sun XF, Liu HR, Feng RE, Peng M, Hou XM, Wang P (2016). Biopsy-proven IgG4-related lung disease. BMC Pulm Med.

[23] Ito S, Ko SBH, Morioka M, Imaizumi K, Kondo M, Mizuno N (2012). Three cases of bronchial asthma preceding IgG4-related autoimmune pancreatitis. Allergol Int.

[24] London J, Martin A, Soussan M, Badelon I, Gille T, Uzunhan Y (2015). Adult onset asthma and periocular xanthogranuloma (AAPOX), a rare entity with a strong link to IgG4-related disease: an observational case report study. Medicine (Baltimore).

[25] Flament T, Marchand-Adam S, Gatault P, Dupin C, Diot P, Guilleminault L (2016). What are the characteristics of asthma patients with elevated serum IgG4 levels?. Respir Med.

[26] Wang XL, Wan J, Zhao L, Da JP, Cao B, Zhai ZG (2019). IgG4-related disease with tracheobronchial miliary nodules and asthma: a case report and review of the literature. BMC Pulm Med.

[27] Wu C, Zeng YP (2020). Mikulicz disease. JAMA Dermatol.

[28] Wallace ZS, Mattoo H, Mahajan VS, Kulikova M, Lu L, Deshpande V (2016). Predictors of disease relapse in IgG4-related disease following rituximab. Rheumatology (Oxford).

[29] Asproudis I, Kanari M, Ntountas I (2020). Successful treatment with rituximab of IgG4-related disease coexisting with adult-onset asthma and periocular xanthogranuloma. Rheumatol Int.

